# Do circadian genes and ambient temperature affect substrate-borne signalling during *Drosophila* courtship?

**DOI:** 10.1242/bio.014332

**Published:** 2015-10-30

**Authors:** Izarne Medina, José Casal, Caroline C. G. Fabre

**Affiliations:** Department of Zoology, University of Cambridge, Downing Street, Cambridge CB2 3EJ, UK

**Keywords:** Behaviour, Circadian, Courtship, Period, Substrate vibrations, Temperature

## Abstract

Courtship vibratory signals can be air-borne or substrate-borne. They convey distinct and species-specific information from one individual to its prospective partner. Here, we study the substrate-borne vibratory signals generated by the abdominal quivers of the *Drosophila* male during courtship; these vibrations travel through the ground towards courted females and coincide with female immobility. It is not known which physical parameters of the vibrations encode the information that is received by the females and induces them to pause. We examined the intervals between each vibratory pulse, a feature that was reported to carry information for animal communication. We were unable to find evidence of periodic variations in the lengths of these intervals, as has been reported for fly acoustical signals. Because it was suggested that the genes involved in the circadian clock may also regulate shorter rhythms, we search for effects of *period* on the interval lengths. Males that are mutant for the *period* gene produced vibrations with significantly altered interpulse intervals; also, treating wild type males with constant light results in similar alterations to the interpulse intervals. Our results suggest that both the clock and light/dark cycles have input into the interpulse intervals of these vibrations. We wondered if we could alter the interpulse intervals by other means, and found that ambient temperature also had a strong effect. However, behavioural analysis suggests that only extreme ambient temperatures can affect the strong correlation between female immobility and substrate-borne vibrations.

## Abstract (precis)

**Summary:** We studied the effects of circadian genes and ambient temperature on the substrate-borne signals produced by fruit flies during courtship.

## INTRODUCTION

Animal communication relies on the accurate production of a signal by one individual which another is able to sense, understand and respond to. Animals communicate by means of their visual, olfactory, hearing, tactile and gustatory senses (Gillam, 2011). Often the signal is encoded in a recurring rhythmical pattern (Gerhardt and Huber, 2002; Hill, 2008; Williams, 2004). The physical properties of the signal (such as its frequency, amplitude, the intervals between its pulses, the length of the bouts, etc) may convey individual and species-specific information from one individual to its prospective partner. For example in the songs of both birds and crickets, the signal waveforms carry information that is important to social behaviour, particularly courtship (Gerhardt and Huber, 2002; Williams, 2004).

During *Drosophila* courtship, the male and the female communicate by several means, including two types of rhythmical signals (Fabre et al., 2012; Mazzoni et al., 2013; Spieth, 1974):

i. Air-borne sounds are produced by the male as he flutters his wing (‘fluttering’); these include a series of pulse and sine songs which are heard by the female. These signals convey information that allows the female to become receptive and to recognise a male of the same species (Ewing and Bennet-Clark, 1968; Ritchie et al., 1999; Tauber and Eberl, 2003).
ii. Substrate-borne vibrations that are generated by repetitive up-and-down ‘quivering’ of the male abdomen at a frequency of 4-6 Hz. Only recently identified, these vibrations appear to travel through the ground towards the females. They correlate with female immobility strongly, suggesting that the female stops moving as a response to the vibrations (Fabre et al., 2012). This behaviour of the female is important as it leads to and allows copulation. It is not known which information-bearing properties of the vibratory signals are processed by the female, and lead to the modification of her behaviour (e.g. her immobility).

The behaviour and physiology of flies, as well as most animals and plants, was shaped, throughout evolution, by their exposure to daily alternations of light and darkness (Zhang and Emery, 2012). These and other daily changes regulate biological processes that occur with a period of around 24 h, and hence such processes are called circadian rhythms. Circadian rhythms are achieved with the help of environmental cues (zeitgebers) plus an endogenous clock that can sustain its rhythmicity independently of the zeitgebers. Zeitgebers entrain the clock so that the cycles remain constant (Zhang and Emery, 2012). This clock depends on genes in a circadian pathway that are rhythmically expressed, giving rise to oscillating levels of RNA and proteins (Zhang and Emery, 2012). In *Drosophila*, the founding member of the circadian gene family is *period* (*per*). Zeitgebers reset the clock by regulating the oscillations of the circadian molecules as well as the interactions of Period with other circadian proteins (Tataroglu and Emery, 2014). Thus, the clock helps flies to optimise their behaviour over the daily cycle (Tataroglu and Emery, 2014): For example, *Drosophila melanogaster* are more active at dusk when they look for food, but reduce their activity when the sun is highest, probably to remain in the shade and avoid desiccation (Pittendrigh, 1993; De et al., 2013). *D. melanogaster* flies also show a daily rhythm of mating activities; they tend to mate more frequently early in the morning and in the mid-afternoon (Sakai and Ishida, 2001; Fujii et al., 2007).

Several studies asked whether circadian genes could also regulate shorter biological processes, such as behaviours associated with courtship, with durations or rhythms ranging from seconds to minutes. Results were mixed: for example, the length of *Drosophila* male courtship was shown to be independent of genes of the circadian pathway, while mutations in the same genes were reported to affect the duration of copulation (Beaver and Giebultowicz, 2004; Roche et al., 1998). More surprisingly, these mutations were reported to alter a particular feature of the courtship song made when the *Drosophila* male flutters his wing: The durations of the successive time intervals between the pulses of the song were reported to fluctuate rhythmically above and below the mean duration. These fluctuations occur over time with a period of 55 s in *D. melanogaster* and may be important for the female receptivity to the signal (Kyriacou and Hall, 1980). Mutations in the circadian genes were reported to affect the period of these fluctuations (Kyriacou and Hall, 1980). However, this result was challenged (Crossley, 1988; Ewing, 1988; Stern, 2014; Arthur et al., 2013), debated and defended (Ewing, 1989; Kyriacou and Hall, 1988; Kyriacou et al., 1990b; Alt et al., 1998).

Here, we investigated the time intervals that occur between the pulses of the substrate-borne vibrations generated by male abdominal quivering to see if they may be important for communication during courtship. We did not find evidence for any cyclic variations in the length of the intervals between successive vibratory pulses over time. Experiments using *per* mutant males and males treated with constant light suggested, however, that components of the circadian pathway and the clock's entrainment by light could alter the mean durations of the intervals *per se*. We found that low and high temperatures also had a strong effect on interval durations. Behavioural analysis whereby we monitored the male courtship and the female's mobility response showed, however, that only large variations in ambient temperature could impair the strong association between male quivering and female immobility, but that an altered circadian clock did not.

## RESULTS

### Analysis of the interpulse intervals of the substrate-borne vibrations during courtship and examination of *per* mutant alleles

#### Substrate-borne vibrations generated by the quivers of wild-type males: are there rhythmical fluctuations in the values of their interpulse intervals?

**Fig. 1.**
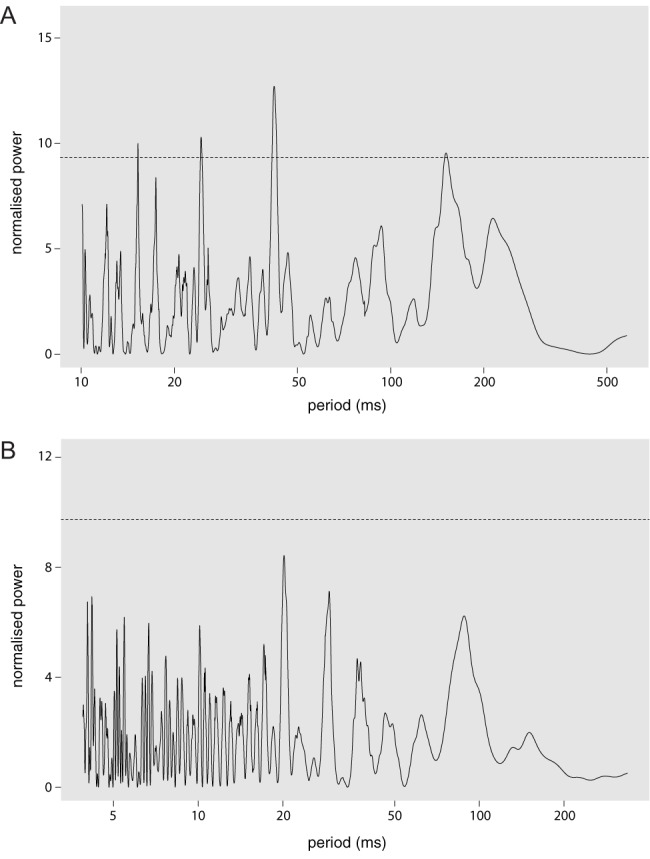
**Lomb–Scargle periodograms of interpulse interval time series extracted from successive bouts of substrate-borne vibrations generated by the abdominal quivering of males during courtship.** Horizontal dotted lines indicate significance values equal to 0.01. Recording time is 600 s in both panels. (A) The periodogram displays three low peaks but nevertheless significant in the range of approximately 15 to 50 ms. The highest peak around 50 ms may represent the fact that quivering bouts are often repeated with that frequency (not shown; our own observations). (B) A periodogram produced with data from another mating pair do not display any high frequency peak. The raw data used for this analysis is available in the supplementary information (files ‘periodogram A’ and ‘periodogram B’). It shows, for each recording, the time point for each vibratory pulse of the series and the IPI values between each pulse.

During courtship, each bout of male abdominal quivering generates a bout of substrate-borne vibrations. A bout of vibrations consists of a chain of pulses, and the interval between two pulses is called an interpulse interval (IPI) (Fabre et al., 2012). It is not known which features of the vibrations generated by the quivers may convey information to the female and the duration of IPIs is a good candidate. We wanted to know if the vibratory signals display a similar periodicity in their IPI durations as that described for the song. Wild-type males were paired with wild-type females. We used laser vibrometry to detect the ground vibrations generated by the male quivering in consecutive bouts during courtship. For every quivering bout, we collected the time point of each vibratory pulse, as well as the IPI values between each pulse and the previous one. We applied the robust Lomb–Scargle periodogram analysis to search for signs of periodicity in our IPI data (Ruf, 1999). In this type of analysis, the presence of peaks of frequencies of high significance reflects cyclical features of the data. However, the peaks we obtained for some recordings were either low or were not present in other recordings. Two such Lomb–Scargle periodogram analyses are showed in Fig. 1. In summary, we were not able to detect any particular pattern of periodicity in the durations of the IPIs in the substrate-borne vibrations.

#### Analysis of the interpulse intervals of the substrate-borne vibrations generated by the quivers of *per* mutant males during courtship with wild-type females

We did not identify any rhythmical fluctuations in the lengths of the IPIs, but it is possible that the *period* gene might influence the mean IPI length *per se*. Males carrying different *per* alleles were paired with wild-type females. We used two different wild-type strains Canton S (Cs) and OregonR (OrR) (both with an endogenous circadian rhythm of 24 h cycles), *per^L^* (28 h cycles), *per^S^* (19 h cycles) and *per^01^* (arrhythmic in laboratory conditions) (Konopka and Benzer, 1971). All types of *per* mutants we observed (*per^S^*, *per^L^* and *per^01^*) performed bouts of quivering.

We found that Cs control males displayed values of IPI durations with a distribution and average similar to those of the *per^L^* mutants (Table 1, Fig. S1A). Note that this was also similar to OrR (Table 1). However, the values obtained for the *per^01^ and per^S^* mutants were significantly different from Cs (Table 1). *per^S^* IPI durations were much shorter than the wild type (23% shorter than Cs; Table 1). *per^01^* IPI durations were only around 5% shorter than those of Cs, an effect much smaller to that observed for *per^S^* (Table 1); the durations of *per^01^* IPIs were similar to OrR (Table 1).

**Table 1.**
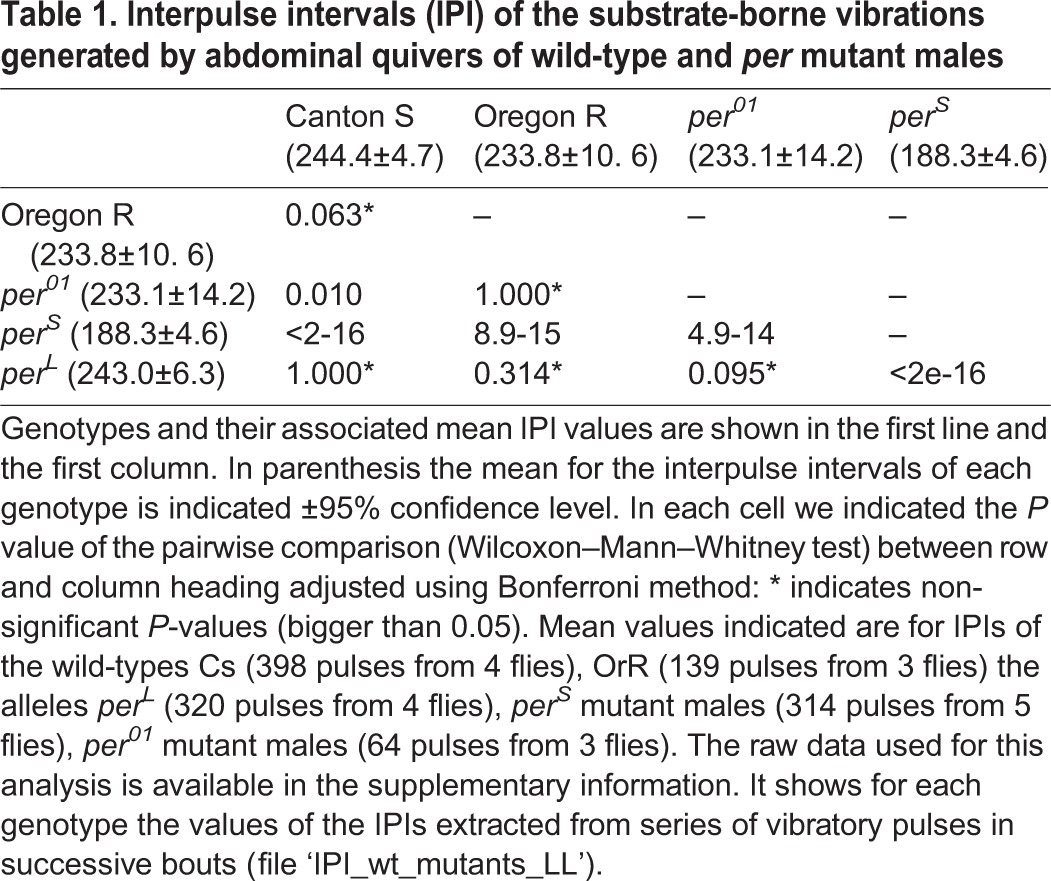
**Interpulse intervals (IPI) of the substrate-borne vibrations generated by abdominal quivers of wild-type and *per* mutant males**

Our results are perplexing: not all the *per* alleles tested resulted in significant effects on the IPIs of substrate-borne vibrations generated by male quivering. Also, some alleles did not modify the IPIs as would be predicted by reports on other behaviours (*per^L^*, *per^01^*; Table 1) (Konopka and Benzer, 1971; Kyriacou and Hall, 1980; Kyriacou et al., 1990a; Andretic et al., 1999; Hamblen-Coyle et al., 1992). However, statistically significant differences do exist in two circadian alleles compare to wild type (*per^S^*, *per^01^*; Table 1).

### Analysis of the courtship behaviours of *per* mutant males paired with wild-type females

**Fig. 2.**
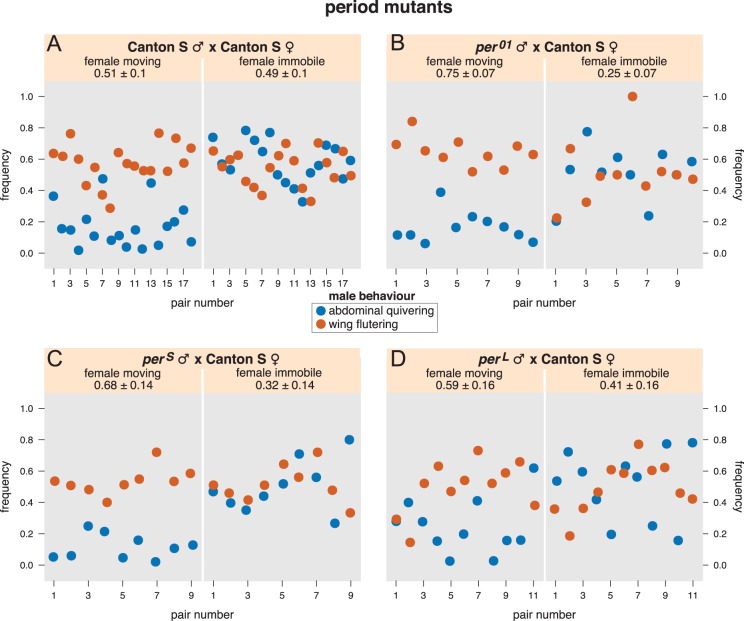
**Two signal-producing behaviours of wild-type Canton-S males and *per* mutant males relative to whether the wild-type female is moving or immobile.** Frequencies were extracted from the ethograms built from movies of courting pairs. The *y* axis shows the percentage of the time the males display wing fluttering (including wing extension/vibration and scissoring) or abdominal quivering. Both behaviours are showed as percentage of the time the female is moving (left) or immobile (right). (A) 18 pairs of Canton-S flies, (B) 9 pairs of *per^01^* male and Canton-S female, (C) 9 pairs of *per^S^* male and Canton-S female, and (D) 11 pairs of *per^L^* male and Canton-S female. Note that each male behaviour is shown without indicating whether the male is performing the other behaviour at the same time. Therefore, reference to Fig. 3 is needed to observe the break down of male behaviours further.

Do the differences in IPI durations identified in the vibrations generated by the male quivering of some *per* mutant males affect courtship behaviour? Males carrying the different *per* mutations were paired with wild-type females and recorded using high-speed high-resolution video imaging. The behaviours of both males and females were annotated and assessed for several behaviours, including male wing fluttering alone, male quivering alone, male wing fluttering with simultaneous male quivering, as well as whether females were moving or stationary. Ethograms of these behaviours were built and used for the analysis. We observed, as we have shown previously, that in wild types, males flutter their wings about as often, independently of whether females are stationary or moving, but male abdominal quivering is strongly correlated with female immobility, which may signal her acceptance of copulation (Ferveur, 2010; Fabre et al., 2012) (Fig. 2A, Fig. S2A).

Wild-type Cs flies are used for comparison with the circadian mutants (See materials and methods). First, looking at female movement during courtship, all the ethograms of *per* mutants showed a decrease in overall female immobility (i.e. females moved more) compared to Cs (Fig. 2). Female immobility varied from about 50% of the courtship time when paired with a wild-type male, to about 30% when paired with a *per^S^* male (i.e. a decrease of around 35%) (Fig. 2A,C). The lowest value was obtained for *per^01^* with a decrease in overall female immobility of around 49% compare to wild-type (Fig. 2A,B). However, both genotypes still showed a high temporal correlation between female immobility and male quivering, as is found with wild-type pairs (Fig. 2A-C). In the case of *per^L^* (which generates IPIs with durations similar to the wild type, Table 1) a few movies did not display a high temporal correlation between female immobility and male quivering. However, most of the other movies did (Fig. 2A,D).

**Fig. 3.**
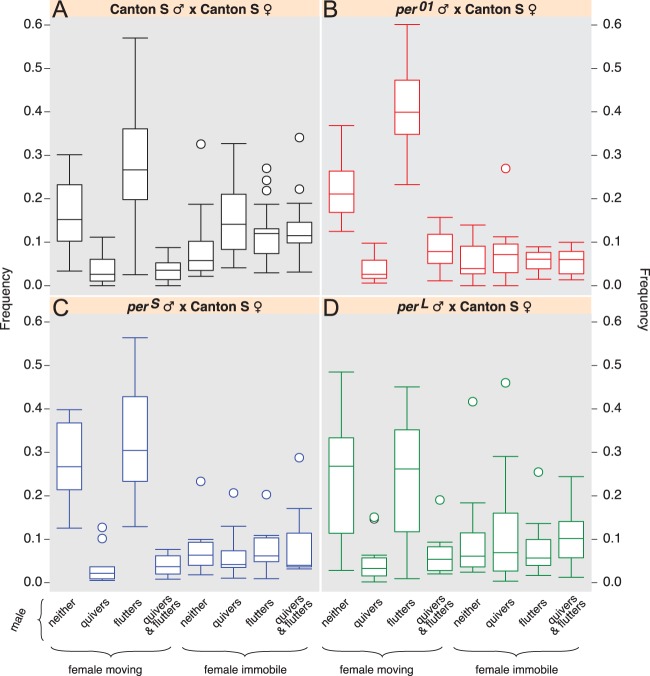
**Male and female behaviours during courtship.** Frequencies were extracted from the same ethograms as those analysed in Fig. 2. (A-D) Boxplots of the frequencies of the analysed behaviours (*x* axis) relative to the courtship time. Circles are the outliers. Results are shown for pairs of Canton-S flies (A), pairs of *per^01^* male and Canton-S female (B), pairs of *per^S^* male and Canton-S female (C), and pairs of *per^L^* male and Canton-S female (D). The raw data used for this analysis is available in the supplementary information (file ‘behaviour_genotype’). It shows, for each movie recorded, the successive behaviours displayed by the fly pair during courtship.

We reasoned that the decreased immobility of the wild-type females when paired with *per* mutants could be explained in two ways: (i) wild-type females may be generally less receptive to these males because other mating cues are altered in *per* mutants, and the females move away from them more; (ii) Mutant males may quiver less often. Our data suggests that both occur:

i. Two simultaneous behaviours of the male and the female occur more frequently when we use *per* mutants males compare to control mating pairs: ‘female moving plus male fluttering’ (especially when *per^01^* mutant males court the females), and ‘female moving plus male neither quivering nor fluttering’ (Fig. 3). This suggests that these males are less attractive to wild-type females, perhaps in part because *per* mutants sing abnormally (Kyriacou and Hall, 1980). As a result the female moves away more.
ii. The high temporal correlation between female immobility and male quivering of *per* mutants (Fig. 2) is confirmed by the fact that ‘female moving plus male quivering’ simultaneously happening is rare (Fig. 3). The behaviour ‘female moving plus male both quivering and fluttering’ is also infrequent (Fig. 3; although this combination is slightly increased in the case of *per^01^*). Yet, the combination ‘female immobile plus male quivering’ is less frequent (Fig. 3). The only way to explain this set of results is if all *per* mutant males quiver less, but the females are immobile whenever the males quiver.

These changes in the attractivity and the quivering of the males may be caused by the genetic background of the *per* alleles or to disturbing *per* function itself.

To investigate if the ethograms still incorporated subtle differences between the genotypes, we tried to find if the data could be subdivided into different clusters. We built a dendogram taking into account all the behaviours assessed, and with each point representing one courting pair (Fig. S1B). If the data were to cluster, this should mean that the samples are different. Visually we cannot detect an obvious clustering of the behaviours by genotype. Quantitative silhouette analysis (Rousseeuw, 1987) also confirmed that none of the data formed clusters: indeed, no significant clustering was observed between any of the genotypes tested (Fig. S1C).

In summary, it appears that despite the significantly different IPI values obtained with *per^S^* and *per^01^* mutants (around 23% and 5% lower, respectively, when compared to Cs; Table 1), *per* genotypes and the significant IPI variations they induced did not modify the strong temporal association between male quivering and female immobility (Figs 2, 3 and Fig. S1B,C). This result was confirmed by robust analysis using clustering and quantitative silhouette tests.

### Analysis of the interpulse intervals of the substrate-borne vibrations generated by wild-type males raised in constant light conditions, and assessment of their courtship behaviours

A consistent effect of *per* alleles on the durations of the IPIs of substrate-borne vibrations was not found. Therefore in an attempt to clarify, we examined courtship of wild-type Cs males maintained under constant light conditions (L:L) for 4 consecutive days, a treatment which is expected to disrupt the clock and behavioural rhythms in a manner similar to the *per* null mutation (Baylies et al., 1987; Pittendrigh, 1981; Price et al., 1995; Zerr et al., 1990). We paired these males with Cs females raised in normal L:D conditions. We found that the IPI values in the vibrations produced by the quivering of Cs males kept at constant light differed significantly from those of Cs raised under normal L:D conditions (Table 2; mean IPI of Cs L:L was on average 10% lower than that of Cs L:D). Note also that Cs constant light IPIs were similar to the values obtained with *per^01^* mutants (Table 2). This result suggests that there might be an effect of the clock on the IPIs of the vibrations generated by quivers.

**Table 2.**
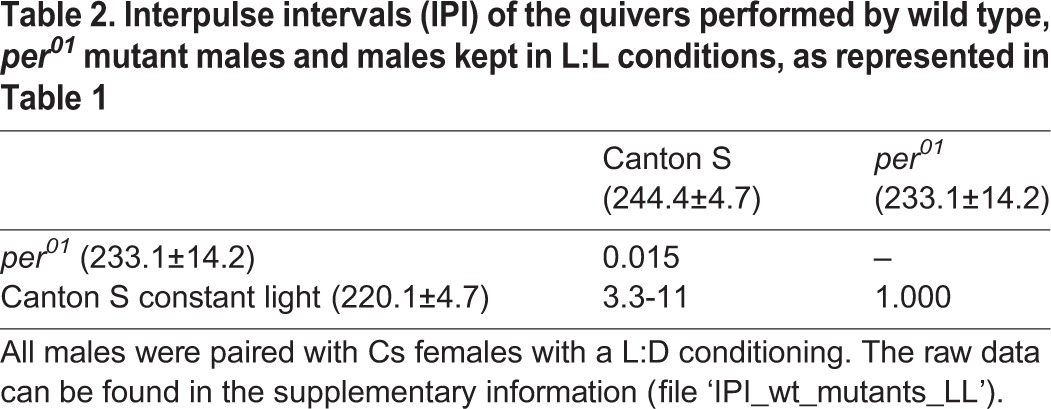
**Interpulse intervals (IPI) of the quivers performed by wild type, *per^01^* mutant males and males kept in L:L conditions, as represented in Table 1**

Next, we looked at the behaviour of these courting pairs. Contrary to what we observed with the *per* mutant males (in particular *per^01^*), conditioning the males in constant light did not induce a decrease in the immobility of the female courted, nor any significant decrease of male quivering during courtship (Fig. 2, Fig. S2B,D). This favours the hypothesis that these behavioural effects observed in *per* mutants are indeed due to the genetic background of the males carrying the *per* alleles or to an independent role of *per* rather than the role of *per* within the clock. We did not observe significant differences between simultaneous male and female behaviour at L:L compare to L:D (Fig. S2A,D; compare with Fig. 2A and Fig. 3A). In conclusion, the effects of the clock and its entrainment by light on the durations of the IPIs in the substrate-borne vibrations is not of much significance behaviourally.

### Analysis of the effects of ambient temperature on substrate-borne vibrations and courtship

**Fig. 4.**
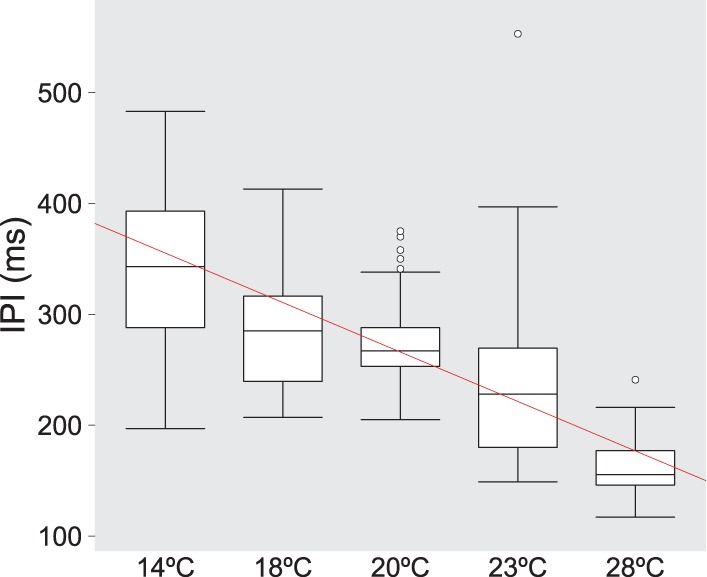
**Temperature effect on the Interpulse Intervals (IPIs) of vibrations generated by abdominal quivering of males during courtship.** Boxplots of IPIs as a function of temperature in the quivering bouts of wild-type Oregon R males paired with Oregon virgin females. The regression line is in red. Data are shown for 2-3 individuals for each different temperature tested (45, 24, 87, 139 and 92 pulses recorded, respectively). The raw data can be found in the supplementary information (file ‘IPI_temperatures’).

We asked if we could modify the duration of the IPIs in the vibrations by using other means. For example, the frequency of the wing beat of insects is temperature-dependent (Walker, 1975). We therefore investigated whether the vibrations generated by male abdominal quivering varied with ambient temperature and also monitored the female's response. Pairs of OrR wild-type flies were observed at temperatures ranging from 14°C to 28°C and showed that the males quivered at all these temperatures. The mean duration of IPIs decreased with increasing temperature in a linear fashion (Fig. 4). IPI durations varied from an average of 349 ms at 14°C to 161 ms at 28°C (Fig. 4; as compared to around 234 ms at 23°C).

**Fig. 5.**
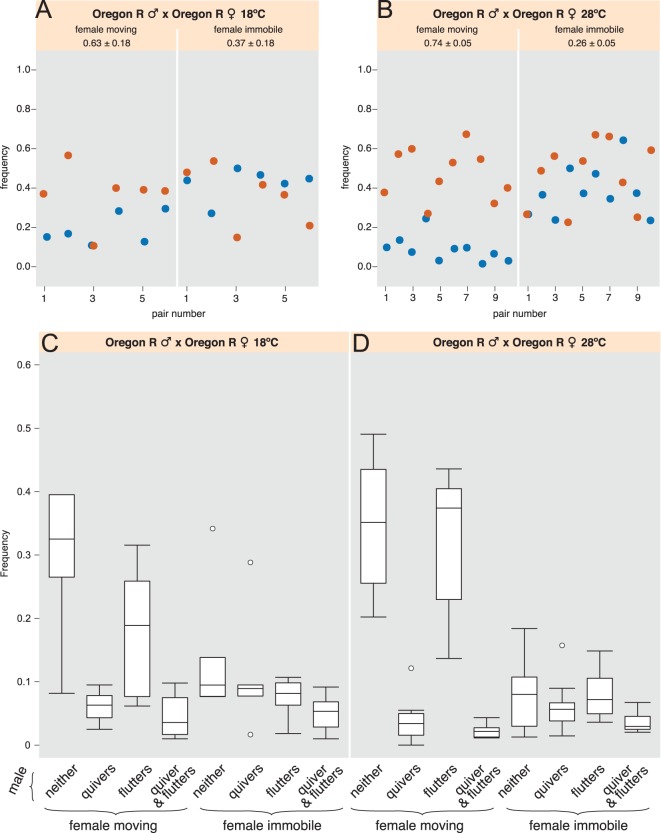
**Behaviours of the wild-types Oregon-R at two temperatures.** (A,C). 6 pairs of Oregon-R filmed at 18°C as represented in Fig. 3. The raw data of this analysis is available in the supplementary information (raw data file ‘behaviour_or18’). (B,D). 10 pairs of Oregon-R filmed at 28°C as represented in Fig. 3 (raw data file ‘behaviour_or28’).

Next, we observed the courtship of OrR at 18°C and 28°C and we compared it to 23°C (Fig. 5A,C, compare to Fig. S2A,C). The level of female immobility during courtship was similar at 18°C and 23°C (Fig. 5A, Fig. S2A). However, we observed decreases both in the correlation between female behaviours and male fluttering (of about 30%), and in the correlation between female immobility and male quivering (about 40%) (Fig. 5A, Fig. S2A). Besides, the simultaneous behaviour ‘male quivering plus female immobile’ was less frequent (a decrease of about 40%), while the behaviour ‘female moving plus male quivering’ increased by 60% (Fig. 5B, Fig. S2C). At 28°C, the female was moving about 20% more than at 23°C (Fig. 5B, Fig. S2A). Male fluttered their wings about as often as at 23°C, independently of whether females were stationary or moving (Fig. 5B). However, at this high temperature, male quivering was poorly associated with female immobility (a decrease of around 40%; Fig. 5B, Fig. S2A). The behaviour ‘male quivering plus female immobile’ was decreased by more than 60% (Fig. 5D, Fig. S2C). All the other relative behaviours were very similar to those observed at 23°C (Fig. 5D, Fig. S2C). At both temperatures tested, we observed similar but stronger effects when looking at Cs wild types (Fig. S3, distribution of IPI values not shown). We conclude that both 18°C and 28°C disturb the courtship of the fly pairs and knock down the temporal association between male quivers and female immobility.

## DISCUSSION

### Lack of evidence for cyclical interpulse intervals in substrate-borne vibrations

The acoustic pulses in the song generated by the *Drosophila* male wings are characterised by the mean value of their IPIs (Bennet-Clark and Ewing, 1968, 1969; Ewing and Bennet-Clark, 1968). In addition, it was reported that the lengths of each consecutive IPIs varied cyclically over time around this mean with a wave length of about 55 s in *D. melanogaster* (Kyriacou and Hall, 1980), although this has been questioned (Crossley, 1988; Ewing, 1988; Stern, 2014; Arthur et al., 2013). Playback experiments suggested that the properties of this fluctuation could influence the success and speed of female mating (Kyriacou and Hall, 1982, 1980). In the case of the substrate-borne vibrations produced during fly courtship, we did not find any such periodicity in the durations of successive IPIs over time. Although we performed a detailed analysis, it remains possible that our recordings were too short to provide enough data points to detect such fluctuations: our flies usually copulate within 10 min. However, even if a periodicity were to exist but were longer than the duration of courtship, it is difficult to imagine how this could convey information to the female, and it would therefore be behaviourally irrelevant.

### The circadian clock has input into the duration of the interpulse intervals but does not alter the temporal correlation between female immobility and male quivering.

Many animals use the durations of the IPIs *per se* for courtship signal recognition and for other types of communication (Bennet-Clark and Ewing, 1969; Gerhardt and Huber, 2002; Hill, 2008, 2009). For example, it is one of the most important acoustic criteria used by female crickets and the planthoppers *Nilaparvata lugens* as they discriminate between the songs of different males (Doherty, 1985; Doherty and Storz, 1992; Shaw and Herlihy, 2000; Gerhardt and Huber, 2002; Ichikawa and Ishii, 1974). Modifications in the durations of the signal IPIs within the song can jeopardise the communication between two potential mates (Gerhardt and Huber, 2002; Bennet-Clark and Ewing, 1969). We found the IPIs characteristic of the substrate-borne vibrations generated by male quivers were altered by mutations in the *per* gene. Vibrations with shorter IPIs were produced by two of the three mutant alleles tested (*per^01^* and *per^S^*). The lack of phenotype and the heterogeneous behaviours observed with another allele, *per^L^*, could be due to other unspecified courtship defects associated with this allele, as was previously suggested (Greenacre et al., 1993).

The *per^S^* allele shortened the duration of IPIs, as might have been expected from its effects on the circadian period (Hamblen-Coyle et al., 1992; Kyriacou et al., 1990a; Konopka and Benzer, 1971; Marrus et al., 1996). The effect produced in *per^01^* males, however, was not as large as would be expected – it is arrhythmic in laboratory conditions (Konopka and Benzer, 1971). But, the substrate vibrations generated by *per^01^* male quivers were not arrhythmic. The IPIs were shorter than the wild type, perhaps fitting with the shorter rhythmical cycles found in the song of some of the *per^01^* mutant flies (Kyriacou et al., 1990b). Treating wild-type males with constant light for several days before mating induced a similar reduction of the IPI durations (Table 2). This is pertinent because both the *per^01^* allele and constant light treatment inactivate the clock (Pittendrigh, 1981; Power et al., 1995; Price et al., 1995; Zerr et al., 1990; Baylies et al., 1987). However, when we studied courtship behaviour, we found that these modifications of the IPIs were not associated with any reduction in the correlation between female immobility and the substrate-borne signal. This suggests that the vibrations generated by the quivers of males with an abnormal clock are fully functional.

### How might the clock and light/dark cycles have some input into the IPIs of the substrate-borne vibrations generated by the male quivers?

The clock genes are known to regulate daily cyclical behaviours in *Drosophila*. These include adult eclosion (Myers et al., 2003), response to olfactory signals (Krishnan et al., 1999), rest-activity cycles (Hall, 2003) and mating receptivity (Sakai and Ishida, 2001). Clock genes may also contribute to the timing of shorter biological processes such as sleep length (Hendricks et al., 2003; Shaw et al., 2002), the timing of feeding (Xu et al., 2008), cocaine sensitisation (Zann, 1984), giant fibre habituation (Megighian et al., 2001), and the length of development (Kyriacou et al., 1990a). In addition, they have been implicated in regulating aspects of courtship such as the duration of copulation (Beaver and Giebultowicz, 2004) and the pattern of the wing song (Kyriacou et al., 1990b; Kyriacou and Hall, 1980). Some of these roles, in particular the two latter ones, are independent of changes in the light/dark cycle (Beaver and Giebultowicz, 2004; Kyriacou and Hall, 1980), and therefore may involve tissues in adult males where the expression of *per* is not light sensitive (Beaver and Giebultowicz, 2004). In the case of the substrate-borne vibrations produced by male quivering, we find that both physiological disruption of the circadian mechanism and genetic disruption of clock genes cause significant variations in their IPIs. It is possible that these effects stem from pleiotropic defects in male fitness, as was reported for the light-dependent effects on male fertility and sperm release abilities of several loss-of-function clock mutations (Beaver et al., 2002).

### Effect of ambient temperature on the durations of the IPIs of the substrate-borne vibrations and on courtship in D. melanogaster

We found that courtship is modified both at low and high temperature, with the intervals between quivering pulses increasing significantly with decreasing temperatures. Our results suggest that, at 18°C, the female is less receptive to any signal generated by the male, including both wing fluttering and abdominal quivering. It could be that the female sensory organs are less efficient at low temperature as, for example, crickets that are deaf at very low temperature (Baden and Hedwig, 2010). However, the substrate-borne vibrations produced by male quivering at 18°C are around 20% longer than the wild type at 23°C. It is therefore also possible that the females are not able to recognise or respond if the ground vibrations that reach them have such long IPI durations. At high temperature (28°C), substrate-borne signals do not correlate well with female immobility either (but slightly better than at 18°C, Fig. 5B). This is associated with a decrease of around 30% in the average IPI duration of ground vibrations. This is the largest decrease in IPI duration observed in this report. Again, this result may indicate that vibrations in which IPIs have such small length do not deliver a reliable signal to females. In addition, the females move more at 28°C (an increase of around 20%) compare to at 23°C. It is likely that, at 28°C, both fly partners move more as high temperature should induce locomotion (Abdullah, 1961). These effects of temperature are also in accordance with previous reports showing that mating success is reduced to 80% at 18°C and continues to decrease until 12°C when it is close to nil (Atlan et al., 1976; Miquel et al., 1976; McKenzie, 1975; Parsons, 1978a,b). Similarly, OrR flies have 55% of mating success at 28°C (Miquel et al., 1976), the hyperactivity of both sexes probably reducing the opportunities for copulation. *D. melanogaster* was originally tropical but has expanded to temperate zones (Schnebel and Grossfield, 1984). The interval 18-28°C may relate to the preferred ambient temperature of *D. melanogaster* for breeding, at which it would be expected that communication would be optimal to induce copulation.

### How could substrate-borne vibrations function in flies?

In all the *Drosophila* species examined, the male quivers during at least one third of the duration of the courtship and temporal coincidence was found between substrate-borne vibrations and female immobility (Fabre et al., 2012). Several physical properties of the vibratory signals generated by the male quivers could convey information to the female during that time. For example, the number or the length of the bouts can affect mating success in anurans and stoneflies (Gerhardt and Huber, 2002; Zeigler and Stewart, 1985); frequency components and/or the amplitude of a signal mediate song recognition in birds, cricket frogs and some Hemiptera (Williams, 2004; Gerhardt, 1986; Cokl et al., 2000; Gerhardt and Huber, 2002; Stritih et al., 2000). However, because amplitude and spectral properties of the substrate-borne vibratory signals risk being damped or distorted by some substrates (Cocroft and Rodriguez, 2005), it is likely that IPIs themselves do encode some information.

The decrease in the duration of the IPIs in the vibrations generated by *per^S^* mutant males was behaviourally irrelevant, yet these IPIs were longer than those generated by a high ambient temperature (where male quivering correlated poorly with female immobility). The correlation between quivering and female immobility was strongly reduced in low temperatures and long IPIs. We hypothesise therefore that the males from one species may broadcast vibrations with IPI durations of a certain range that are recognised by the females from the same species. For *D. melanogaster*, this range may be localised somewhere between the values obtained at 18°C and 28°C. This would be reminiscent of other insects where temperature contributes to defining the range of discriminable IPIs (Gerhardt and Huber, 2002). Such a strategy would be beneficial for *Drosophila* reproduction as the female receiver should detect and respond to all the conspecific signals likely to be heard over the normal range of breeding temperature (Gerhardt and Huber, 2002). Other *Drosophila* species have been described that broadcast substrate-borne vibrations with interpulse intervals much shorter than that of *D. melanogaster* (Fabre et al., 2012; Mazzoni et al., 2013), thereby improving discrimination between different species.

## MATERIALS AND METHODS

### Mutant and wild-type flies

Flies were raised on standard wheatmeal medium under a 12:12 h light:dark cycle (unless otherwise stated) and kept at 23°C with 65% humidity. For the analysis of wild-type behaviour, we used OregonR (OrR) and CantonS (Cs). *per^01^*, *per^S^*, *per^L^* mutations were made by the late Ronald Konopka and kindly given by Ralf Stanewski (University College London, UK). For details of mutant alleles, see FlyBase (dos Santos et al., 2015). They were generated on a Cs background and we backcrossed them to Cs for two generations. Therefore, we compared the courtship of male mutants paired with Cs females to that of Cs males and females. Adult flies were collected upon eclosion with light CO2 anaesthesia. Before mating, individual males and small groups of five to ten virgin females were kept isolated in vials with fresh food. For laser vibrometry experiments, wings were cut so as to reduce noise in the recordings. Unless otherwise stated, courtship was filmed and laser vibrometer was performed (including the experiments performed at constant light) at a temperature of around 23°C.

### Recording vibrational signals with laser vibrometry

Video and laser vibrometer recordings were conducted on a vibration-damped table in a soundproof room. Flies were placed into cylindrical chambers of approximately 10 mm in diameter and 6 mm in height, made of resin. The top of this cylinder was a transparent film through which the flies were recorded using the Stingray F-33B camera. One side of the cylinder consisted of a piece of thermal foil, a membrane made of silver metallised polyester material, with an albedo of approximately 0.8 (Sub Zero Technology). The beam of a OFV-534 laser vibrometer (Polytec) was directed perpendicular to the surface of this membrane. Signals were digitised with 12 bit amplitude resolution with a PCI MIO-16-E4 card (Analog Devices) and with LabView (National Instruments) on a PC. Signals were transformed into wav data with the Audacity (http://audacityteam.org) or Neurolab softwares (Knepper and Hedwig, 1997). Video and laser vibrometer recordings were synchronised at the start by brief interruption of the laser path; this produces both a momentary peak in the oscillogram and a black frame in the video. Oscillograms were analysed with the Amadeus Pro (http://www.hairersoft.com/pro.html) and the Raven software (http://www.birds.cornell.edu/raven). Interpulse intervals of the vibratory signals were obtained from these oscillograms. IPIs with durations higher than 600 ms (2-3 times the average IPI) were considered to belong to two different bouts and were not used for quantifications.

It should be noted that our set-up only allows recording the quivers when the male stands on the reflective membrane on which the laser is directed, and not when he stands on the other five sides of the chambers; we will therefore be missing some quivering bouts that are not included in the data record and analysis. For the study of rhythmical fluctuations, we analysed only those recordings where the flies remained more than 90% of the time on the recording membrane (rather than on the other sides of the chambers not targeted by the laser vibrometer), so as to take into account most of the substrate-borne vibrations generated by the male quivers. In any case, the Lomb–Scargle periodogram analysis was used and it accounts for potential gaps in the records (Ruf, 1999).

### Behavioural recording

Pairs of flies were tested in a single trial when they were 4 days old. Their behaviour was recorded with a 103 macro lens and a Firewire Stingray F-033B camera (Allied Vision Technologies) and acquired with Astro IIDC (Aupperle Services and Contracting) into a laptop computer. For analysis of the wild type, 30 courting pairs were recorded and analysed. For other studies, a minimum of 5 pairs of flies was tested. Transparent plexiglass courtship chambers (10 mm diameter and 6 mm height) were assembled from two half chambers each of 3 mm height. Each fly was collected with a mouth aspirator and introduced into one half chamber. After a recovery period of 5 min, both halves were fused, and filming of the pair was commenced. Recording was started at the initiation of courtship and for approximately 600 s, or until copulation occurred. Each pair was tested only once. Before each test, chambers were washed with ethanol and dried.

### Behaviour annotations and analysis

Movies were annotated with the ‘Annotation’ software version 1.3 (http://annotation.en.softonic.com/mac), registering all standard male courting behaviours (such as orientating toward the female, following the female, proboscis extension, licking, tapping), in particular when males showed wing fluttering (this behaviour comprises wing extension/vibration and scissoring) and/or abdominal quivering, and also whether the female was moving or immobile. The data for each movie were imported into Excel files (Microsoft). For statistical analysis and generation of diagrams, we used the R programming language and software environment (http://www.R-project.org). All intervals shown in the paper are for 95% confidence level.

### Raw data

We attach Excel files with the raw data for all our experiments in the supplementary information.
